# Smart E-Textiles: Overview of Components and Outlook

**DOI:** 10.3390/s22166055

**Published:** 2022-08-13

**Authors:** Rebecca R. Ruckdashel, Ninad Khadse, Jay Hoon Park

**Affiliations:** Department of Plastics Engineering, University of Massachusetts Lowell, Lowell, MA 01854, USA

**Keywords:** electronic textile, sensor and actuators, smart textile

## Abstract

Smart textiles have gained great interest from academia and industries alike, spanning interdisciplinary efforts from materials science, electrical engineering, art, design, and computer science. While recent innovation has been promising, unmet needs between the commercial and academic sectors are pronounced in this field, especially for electronic-based textiles, or e-textiles. In this review, we aim to address the gap by (i) holistically investigating e-textiles’ constituents and their evolution, (ii) identifying the needs and roles of each discipline and sector, and (iii) addressing the gaps between them. The components of e-textiles—base fabrics, interconnects, sensors, actuators, computers, and power storage/generation—can be made at multiscale levels of textile, e.g., fiber, yarn, fabric, coatings, and embellishments. The applications, current state, and sustainable future directions for e-textile fields are discussed, which encompasses health monitoring, soft robotics, education, and fashion applications.

## 1. Introduction

The term “smart textiles” has emerged to describe artifacts that interconnect active functionalities (often electronic and computational-based) as a wearable artifact [1,2]. These textiles engage almost all senses—olfactory, visual, auditory, haptic or tactile, and time [3,4]. Smart textiles convert stimuli from the environment (temperature, light, chemicals and moisture, pH) or interactions (mechanical force and electromagnetic field) into responses in aesthetic (color, light intensity, fluorescence, shape or form) or physical (mechanical, electrical, thermal, chemical, wetting or moisture transport) properties [5,6,7]. They are dynamic, biomimetic systems [4,7,8].

In general, smart textiles are composed of a base fabric, interconnects, sensors, actuators, a power source or generator, and a computer processing unit. Although all components can be made from textile materials (polymers, fibers, yarns, fabrics), not all are. This review specifically focuses on electronic-integrated textiles; we point the readers to another review article for non-electronic smart textiles [9]. Smart textiles are classified by obscuring or highlighting their textile and electronic attributes:

(1) Interaction with the environment—passive (sense), active (sense and react), or very smart (sense, react, and adapt) [9,10],
(2) Form, location, or attachment method [11], e.g., “soft systems”,
(3) Components involved and the level of human interaction [12], and
(4) Electronic (electronic textiles or “fibertronics”), which require a computer and batteries, or non-electronic (“reactive”, “self-actuated”, or “adaptive”), which do not [4,7,8,13].

The role of the “textile” in smart textiles has evolved through three generations over the past few decades: (1) rigid electronics on a textile platform, (2) devices embedded in textiles, and (3) fully textile devices [14]. In the first generation, in the mid-1990s, wearable computing, audio processing and signal processing with the textile as a platform were researched. [11,15] In the second generation, in the early 2000s, toolkits democratized prototype development [15] yet kept the textile as a platform. As a result, research moved towards non-electronic or fully textile solutions. In the third generation, the textile has transitioned from being a surface on which components are attached to the interface for human and computer interaction [7]. Thus requiring new modes for handling personal data, technologies to support virtual commerce, and manufacturing processes for mass personalization, and evolving the understanding of a textile’s activities [7].

**Figure 1:**
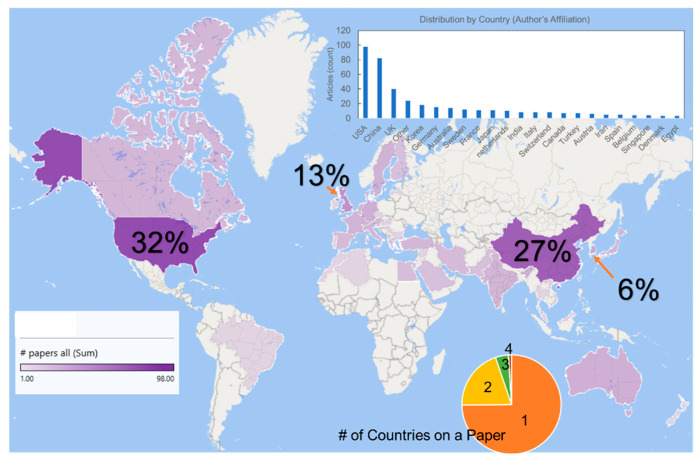
Global representation of 300 scientific articles published over the last 5 years.

The uniqueness of the smart textile field when compared to other materials, is in its highly interdisciplinary and collaborative nature. Figure 1 shows a collective examination of 300 scientific articles published on smart textiles within the last 5 years. The United States lead the number of articles published, followed by other major players such as China, the UK, South Korea, and Germany. It is notable that about 25% of papers are published by two or more countries working together on one paper, which is one indication of the global collaborations of this subject.

**Figure 2:**
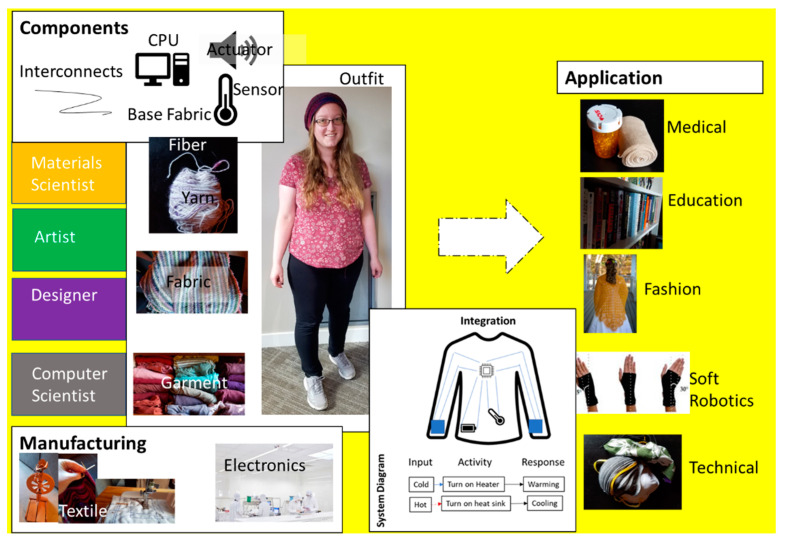
Overview of the current perspective on smart textile. Part of Figure adapted from [20,21].

Such collaboration spans multiple disciplines, components, and scale, as illustrated in the overview of smart textiles in Figure 2. Smart textiles are multifaceted in nature; each component of the e-textile needs to be “woven” into one garment. As also described in Figure 2, an e-textile encompasses the integration of an input, activity, and response, which is realized in the form of a sensor, actuator, interconnects, and power storage/device. The development and incorporation of each component need contributions from seemingly unrelated fields, such as material scientists, computer scientists, artists, and designers.

These collaborations, while enabling the innovation of smart textiles, also present challenges to address gaps brought at every step, from the manufacturing of each component to the end-use application. A comprehensive review article of e-textiles dates back to 2012 [16], while more recent ones have dealt with focused applications [17,18], methods [19], or non-electronic textiles [20]. To date, no comprehensive review that deals with the components of smart textiles and their challenges due to the interdisciplinary nature of this new, fast emerging subject exists. As such, this review aims to holistically examine the roles of each component and the expertise involved for each in a smart textile. We will examine (i) the production and functionalities of each constituent, (ii) a brief overview of applications, and (iii) the current needs and outlook.

## 2. Components of Electronic Textiles

### 2.1. Conductive Materials

Conductive materials are required to make electrical elements such as resistors, capacitors, inductors, and interconnects [22,23]. While flexible electronics can be made by decreasing the electronics’ dimensions, switching to flexible conductive materials allows for long-term adaptation of textiles [24]. Raw materials, yarns, or fabrics can be made conductive, while sensors, actuators, and power components can be constructed by layering conductive fabrics [25]. The method selection depends on the available equipment, desired conductivity, percolation threshold, and fabric rigidity requirements.

The textile can be made conductive at any production step: polymerization, fiber spinning, insertions during fabric construction, or during post-processing such as by coating or printing. Polymerizing conductive polymers or copolymers ensures high compatibility, yet it is costly and may not result in spinnable materials. Conductive additives, such as metals, carbon black, carbon powder, carbon whiskers, graphene, nanotubes, ionic liquids, and conductive polymers, e.g., polyaniline (PANI) and polyvinylidene difluoride (PVDF), can be included during fiber spinning to make an electrically conductive composite fiber [26,27]. However, the percolation threshold, the amount of conductive material needed to form a conducting network, and desired conductivity will impact fiber rigidity. Table 1 below lists the conductive materials used, conductivities, and percolation thresholds. Metallic materials tend to have a lower percolation threshold and higher conductivity than their non-metallic counterparts, so this will limit which of the available conductive materials, metallic or non-metallic, should be used. Additives will make the fabric more rigid since they are less compliant than polymers. Conducting materials that have a wire-like aspect may be added during fabric construction as a weft insertion in knitting or weaving. However, these materials will need to be capable of undergoing the same tension and bending as their fibrous counterparts without breaking. Metallic printing is an exciting way to add conductivity during post-processing, especially with the advent of nanoparticle inks [9] and microdroplet printing [23]. While it may be easiest to add conductivity during post-processing, these materials are more susceptible to cracking, delaminating, and chipping due to poor adhesion and a difference in material rigidity [28,29]. Overall, the expertise and available equipment tend to select the production step for conductivity addition rather than strategic benefit.

### 2.2. Interconnects and Communication

**Figure 3:**
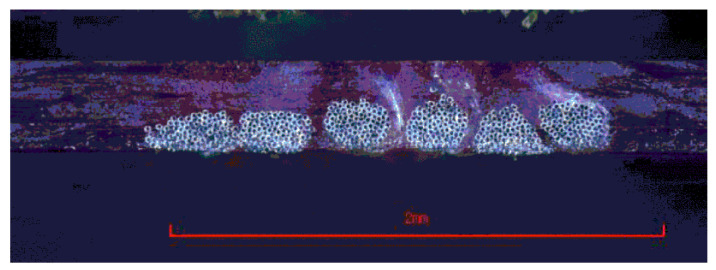
Smart composite made by embroidering transmission lines with conductive bobbin thread into twill woven S-glass pre-impregnated with epoxy resin then consolidating and curing [46]. In the figure, the embroidery thread (six cylinders) is composed of individual Kevlar fibers (navy dots) plated with silver (white). Reproduced with permission from Microwave Theory and Techniques. Copyright 2016 IEEE.

Interconnects, wires, or antennas relay information and power between components, the computer, and the wearer. Wires are manufactured by extrusion processes or embroidered conductive threads, while antennas can be made from conductive threads, embroidering, or fabrics. Wired interconnects both attach items to the textile [15] and conduct electricity for power and data communication between components and the wearer [14,22]. Interconnects must be robust against abrasion, puncture, laundering, and folding; this is necessary to prevent device failure if a line is cut or abraded. Other attachment methods, e.g., hot bar soldering, insulation displacement connections, and anisotropic conductive adhesives, often fall short of meeting the needs of electronic textiles [43] due to corrosion or short circuiting over time [44,45]. The bending rigidity of encapsulate films impacts cracking: higher modulus encapsulate films can be thinner and support positioning of the neutral bending axis at the encapsulate center to prevent stress concentrations and the cracking of thin wires [23]. More recently, conductive inks and threads have been used in place of rigid, soldered, un-washable plastic insulated wires. Sewing, sputtering, soldering, and snaps can be used as interconnects [22]. Sewing or embroidering conductive threads may use bobbin feeding instead of needle feeding depending on the machine thread flexibility; since the bobbin thread undergoes less bending and remains unidirectional in the fabric (Figure 3) [46], flexibility is often assessed by a curl test [47,48].

The dual roles of wires can be devolved into two separate media: one to “attach” components and one to “communicate” power and data. Attachment methods include hook-and-loop fasteners, pockets, elastic material, iron-on (thin film circuit), sewing, and glue [22,49,50]. Wireless communication uses antennas and resonators [11,51,52]. Antennas can wirelessly power inaccessible components, e.g., ingestibles [51]. However, the drawbacks of wireless systems include a slower response time [11], signal degradation [51], bulkier components due to powering needs [53], and proprietary communication protocols [14,53].

### 2.3. Electronic Sensors and Actuators

Sensors can monitor movement, physiology, or the environment. Movement sensors require signal processing whether they are inertial motion, optics, or strain sensors, rigid electronics, or fully textile, piezoresistive or conductive textile (Table 2). Strain sensors convert mechanical deformation into measurable electrical signals and, as with pressure sensors, can be resistive or capacitive [14,54]. Resistive pressure sensors change electrical resistance when stretched or compressed [22]. Capacitive pressure sensors store or release electrical energy. Strain sensors are made with conductive materials by screen printing, sewing, knitting [55], or layering fabrics [14], although, repeated straining of carbon filler–polymer matrix or multilayered systems can cause non-linearity and signal drift, resulting from delamination, which decrease the device lifetime [56,57]. Fiber- or yarn-level devices can be made by combining a conductive component and flexible substrate into a composite or layered structure [56], e.g., from dielectric-coated conductive yarns or piezoresistive materials [14]. Fabric-level devices can be made from conductive threads or sandwich structures. For example, conductive threads have strain dependent resistance due to changes in the effective yarn length when sewn [14] or knitted [58]. A resistive or capacitive sensor can be made from a sandwich structure (conductor–spacer–conductor) [15]. A capacitive sandwich structure (conductor–dielectric–conductor) can be made as a thread or fabric by embroidering, patterning, or laminating electrodes [16,22]. Conductivity in a sandwich structure (fabric–dielectric–fabric) varies depending on the dielectric layer’s thickness [14].

Physiology sensors monitor internal and environmental conditions by way of electrodes, near-infrared spectroscopy, microfluidics, and (Table 2). Some sensors discern multiple stimuli and are called “multimodal”. Sensors are disease agnostic, such that a thermistor embedded in a textile can monitor cardiovascular health, skin ambient temperature, and the foot ulcers or wound infections of diabetic people [65]. Textile-based sensors can diagnose cystic fibrosis based on pH, sodium, conductivity, and hydration levels during exercise [50]; detect immune responses [68]; monitor neurodegeneration [64]; observe babies for poor circulation and heart disease [69]; and sense moisture in wounds, beds, or athleticwear to reduce skin pathologies [25].

Electrodes, an electronic sensor constituent, are conductive contacts between the wearer and a smart textile system. They can monitor or provide feedback, e.g., functional electrical stimulation (FES) [14,70]. Skin irritation, conformability, and discomfort are major concerns for electrodes and their adhesives [24,70]. Electrode placement for an electrocardiogram (ECG) is notoriously challenging and affects reliability [70,71]. Textile-based sensors and electrodes provide useful preventative, early detection, and serious health condition data. All of the aforementioned sensors may be used in conjunction with the actuators to yield responses to e-textiles that engage with human’s senses, e.g., display, mechanical actuation, audio, or combinations therein (Table 3).

### 2.4. Power–Energy Generation and Storage

A smart electronic textile requires power for electronic components throughout the lifetime of the device by the use of batteries or energy generators [11,12,15,51]. Batteries store energy; ideally, batteries would be replaceable and rechargeable [14]. However, conventional batteries tend to be bulky, rigid, and not washable [14]. Thin, flexible, hidden batteries may be made by embroidering or printing with conductive materials [78]; the advancement of nanomaterials may also help with energy conversion efficiencies [79].

Reducing component consumption, through “wake up” and “sleep” functions, and increasing energy efficiency may also extend the battery life and reduce the risk of overheating and burns [11,12]. Harvestable energy sources include light (solar or artificial, “photovoltaic”), human body heat (“thermal”), human motion (pressure or mechanical, “piezo”) or friction (“tribo”), and wind [22,26,27,51]. Not only must the energy source match the power consumed of the device, it must also provide enough current and voltage [51]. Hybrid energy generators increase and stabilize the output for a constant power supply [27,80]. Power can be wirelessly transferred through planar spiral coils embroidered with conductive thread onto a woven polyester glove using inductive coupling [81]. Energy generation stability is degraded by cyclic mechanical loading, chemical treatment, and environmental factors [27]. For example, piezo materials lose their dipoles above the curie transition or melting temperature [27]. Wearable energy generation must be efficient, stable, mechanically durable, and survive scaled-up textile production methods [82].

**Figure 4:**
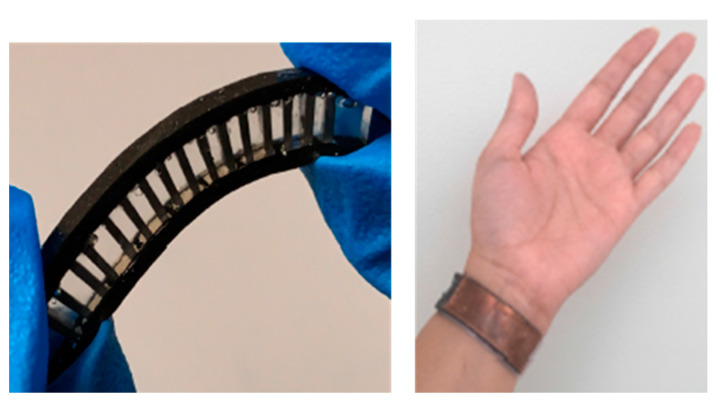
Thermoelectric device with n- and p-type off-the-shelf thermoelectric legs sprayed eutectic gallium indium (EGain) liquid metal for interconnects and encased in PDMS (**left**). Device coated with copper to distribute heat (**right**) [49]. Reproduced with permission from Appl. Energy. Copyright 2020 Elsevier.

While thermoelectric generators can be embedded in fabrics (woven, knits, synthetic, natural fiber), the current thermoelectric generator materials have limited practical use due to being high profile (discomfort), a low temperature differential (output voltage) and their bulkiness (energy generation) [83]. For example, a flexible thermoelectric generator based on the Seebeck effect converts heat lost from the wrist into 35 uW/cm^2^ energy under walking conditions; it was made from a thermoelectric pillar assembly attached to a wristband (Figure 4) [49].

Solar cells can be dye-sensitized, perovskite, or polymer. Dye-sensitized solar cells follow a photosynthesis-like process: incident light excites electrons, from the dye into the semiconductor conductive band, typically titanium dioxide, which generates a current; a redox electrolyte reduces the generated positive charge, or “hole”, by replacing it with an electron [84]. Alternatives to dye-sensitized solar cells using textiles use other photosensitizers in place of dye, including doped polymers or other solids. Perovskite solar cells have much higher conversion efficiencies than dye-sensitized solar cells (29% possible), likely due to a higher charge carrier mobility, long carrier diffusion length, and near instantaneous charge–hole separation (~2 ps), although they require a solid state electrolyte [85]. Scalable methods for textile photovoltaic manufacturing include optical fiber-style thermal drawing with embedded electronics [86,87], wire coating [88], or inkjet printing [4]. For thermal drawn fibers, functionality can be imparted pre-draw (preform assembly) or post-draw (deposition or etching) [87].

Energy from human motion can be collected by triboelectric or piezoelectric methods operating at the pace of human motion, about 1 Hz [89]. Triboelectric energy is collected from mechanical friction between pairs of materials with differing electron affinity in four modes: single-electrode, lateral sliding, vertical contact-separation, and freestanding triboelectric-layer [27]. Triboelectric energy is a natural choice for powering a wind sensor, pedometer, pulse monitor, or sleep monitor [27]. Production methods include a coaxial dielectric/electrode fiber, which is woven or knit, fabric bands woven as strips, spacer fabrics made from 3D weaving or knitting, and layer fabrics [27]. Piezoelectric energy is harvested by converting mechanical to electrical energy, e.g., heart rate, tactile sensing (input), pressure, falls detection on floor [27]. Piezoelectric energy generators have a sandwich structure of a piezo material between two conducting layers with a cotton fabric separator to prevent electric shorting [27]. Piezoelectric polymers, such as PVDF and polyvinylidene fluoride–trifluoroethylene (PVDF-TrFE), and ceramics, such as lead zirconate titanate (PZT), can be combined to improve the piezoelectric constant (more ceramic) and reduce brittleness (more polymer) [27]. Nano-scale piezo materials are sensitive to small forces, while fabrics made from piezoelectric yarns have a higher output than the yarns [27]. Piezoelectric properties depend on the materials and processing.

### 2.5. Computer or Central Processing Unit

The computer or central processing unit, “CPU”, is the brain of the system. Computers operate control systems, process information, and store data on or off garment [11,51].

The logic gates, e.g., transistors, process the information by performing logic operations. Transistors—defined by an electron gate, source, and drain—can be made by attaching traditional elements, soft lithography, or evaporation [22]. Alternatively, logic gates could be made from multistimuli-responsive polymers, although producing an “AND” function requires two different stimuli to produce one response [5]. A computer must be able to handle the amount of data produced by the components (Random Access Memory (RAM) and storage memory) and be updateable. Textile computers have transition states and ambiguity between “1” or “0” [90]. While it is possible to make a textile computer [90], most applications still use a traditional CPU, as with the LilyPad and Adafruit toolkit break out CPUs, or a smartphone device.

### 2.6. General Applications of Electronic Textiles

Electronic textiles continue to garner interest from academia, government agencies, and industry researchers. The following details a handful of the most promising applications of the last decade. For a more detailed review of electronic textile applications, please refer to other excellent review articles [18,19].

Smart textiles are a medium for interactions between humans and computers. These robots “link up human intentions with machine actions” [91]. Soft objects can be enhanced to support interactions. A 3D printed elastomer network, “optical lace,” uses optics to sense deformation [92]. Alternatively, a touch sensor or deformable robot can be made by cutting, layering, and heat bonding conductive fabrics through “3D fabric printing” [93]. Interactive, tactile learning is supported by electronic embroidered books [94], while “sonification” can provide assistive auditory cues or the translation of non-audio data into sound [15]. Even a sound system can be controlled by a textile touch sensor [94].

Patches can be used to control machines. For example, alphabet or coded numeral signals can be relayed wirelessly through triboelectric interaction with a splitting ring structure patch [91]. Alternatively, a four-mode controller can take advantage of clenching motions detected with a PVDF microelectric-mechanical system (MEMS) printed onto artificial skin and attached to the left and right wrist [95].

**Figure 5:**
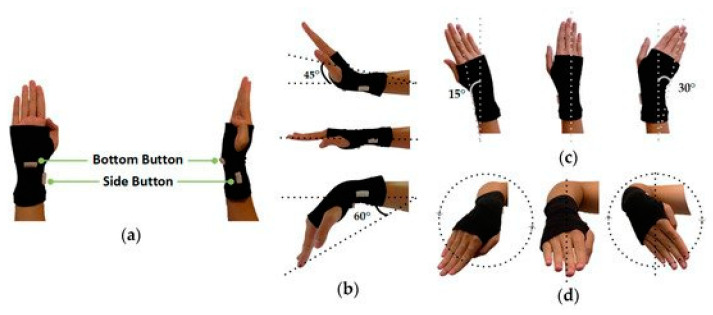
Glove-embedded strain sensor captures gestures for vehicle control: prototype (**a**) and movement in vertical (**b**), horizontal (**c**), left–right (**d**) directions [16]. Licensed under a Creative Commons Attribution (CC BY) license.

Clothing is another useful surface or medium. Glove-based devices can use gestures to control machines [96]. For example, human visual cognitive load and attention switching during driving can be decreased with a gesture-capture glove with embedded strain sensors (Figure 5) [16]. Electronic devices can be controlled by a highly conductive yarn woven into a touch sensitive sleeve with a consistent 95% recognition rate after 30,000 swipes [97]. The sleeves were woven from a highly conductive yarn composed of a copper wire core wrapped in a braided 2-strand silk yarn and coated with polyurethane [36].

**Figure 6:**
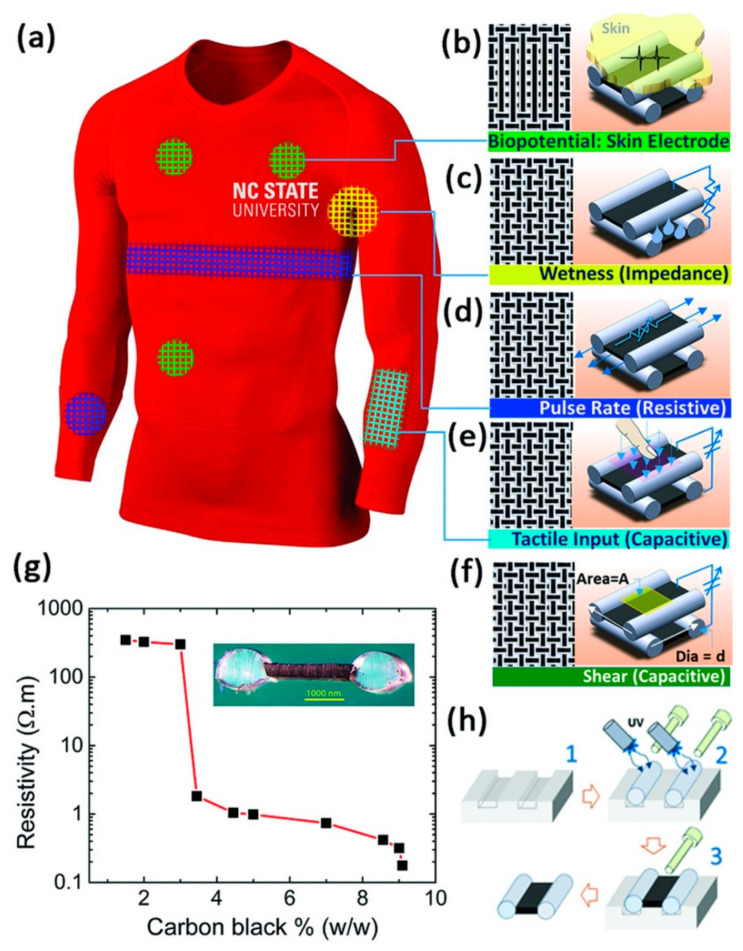
Multimodal sensor schematic (**a**) showing the five modes—biopotential (**b**), wetness (**c**), pulse rate (**d**), pressure (tactile input) (**e**), and shear (**f**)—carbon black percolation threshold ((**g**): inset shows fiber cross-section) and three step fiber casting method (**h**) [100]. Reproduced with permission from Adv. Mat. Tech. Copyright 2018 Wiley.

Standard fiber spinning processes can produce bifunctional actuating/sensing fibers for haptic feedback and user interaction sensing [98]. Alternatively, a co-rolled preform thermally-drawn capacitor fiber can function as a 1D slide sensor (fiber) or 2D touchpad sensor (woven fabric) [99]. A solution cast bicomponent dumbbell-shaped conducting/insulating fiber woven into fabric can respond to five different types of stimuli (Figure 6) [100]. Accounting for time allows a knit capacitive and resistive sensor fabric to distinguish between complex no touch, touch, and metallic touch interactions after signal processing with an Arduino-based program, “Teksig” [101].

Finally, smart textiles can encourage interactions between humans: bridging the gap between interactivity and interconnectivity [4]. For example, a dress that changes color in response to the wearer’s brainwaves [2] can externalize mood and encourage interaction. Sharing a smart textile object can promote social interactions through joint discovery [102]. A gown bodice can recast a wedding ritual as a public sharing and melding of heart beats, “Data Vows” [45]. The bodice was composed of an Adafruit Flora microcontroller, a Polar One Heart Rate sensor, light-emitting diodes (LED), and a Karl Grimm silver conductive thread [45].

## 3. Current Limitations

### 3.1. Wearer Needs

Wearables must be wearable and functional, or “work”. [58]. Appearance, comfort, a light weight, user friendliness, durability, and a long battery life (24 h) or low electrical power consumption are important to wearers [103,104]. They expect continuous connectivity, energy efficiency, data security, and privacy [12]. End users may also have environmental requirements [24,103,104,105], a strong preference about synthetics versus natural fibers [51,106,107], and a desire to have the product stand out or be concealed [1]. The level of sensitivity to design are also specific to demographics, e.g., people with autism tend to be more sensitive to textures, sounds, state cycling, hidden relationships, the poor alignment of visual cues, and physical interaction [102]. Other concerns include pattern reversibility [108], reconfigurability [12,103], interactions [108], game-like elements [14,109], washability [82], and durability [22,103] throughout the product lifetime from the materials’ selection and manufacturing to the device’s use and end of life [13]. Populations have different needs and the conclusions of any user study are not universal [110]. Each end user will have differing preferences, so human wear trials are essential to making an acceptable product. Invoking these opinions before prototyping and throughout development will lead to a better product–market fit and potential commercial success [14]. It is notable that academic research has yet to launch commercially viable smart textiles.

### 3.2. Interdisciplinary Collaborations: United Intention with Divided Focus

Smart textiles’ research is collaborative; yet, fostering collaborations is a challenge [24]. Skills training for new textile techniques, sustainability and ethical requirements for manufacturing, and textile deliverables must be managed [106]. Research refines assertions into accepted facts [111]. Disruptive technology, such as smart textiles, depends on challenging the status quo; however, academic productivity depends on deep research in one area with a track record of publications [12,112]. As a result, researchers tend to rush into “gap-filling” instead of collaborative inquiry [7,12,112].

While interdisciplinary research has become more commonplace, collaborations for smart textiles span a much wider range of disciplines, sectors, and countries; these include scientists, artists, designers, computer experts, technologists, electrical engineers, manufacturers, and wearers in academia, government, and industry [11,15,103,113]. They are united in exploring concepts for smart textiles yet separated in their approach.

On the one hand, scientists discover new materials and characterize their properties, while engineers apply a material’s properties and functionalities to solve problems. On the other hand, designers and artists move materials out of the science lab and into practical applications. Artists question the underlying structures of what exists, how it is made, and who participates in making or using it [90]. Designers learn a material’s uses by experiential tinkering, broader contexts, and collaborative actions through material-based or holistic design processes [4,8,51]; designs are based on form and tangible material aspects, such as exploiting the sidedness and 3D nature of textiles for interactions [101]. Smart textiles may be made from adaptive materials or materials made adaptive through design [8].

Another notable collaboration that profoundly affects smart textile functionality is the interdependence of software and hardware [12]. Data collection [12], conversion to actionable information [24], and on or “off textile” machine learning [14] must all work within the physical limitations of the textile. Smart textiles share sensitive data—biometric, behavioral, work, geolocalization, and mobility [103]—through a smartphone, gadget, website/social media, or ambient display [3]; who has data access must be limited to protect data and privacy [11,24]. Data security, the redundancy and the trustworthiness of a network can be maintained through blockchains, software upgrades, patches, and modifications [11,12].

Finally, the collaboration between textile and material scientists is central to making smart textiles a reality. Material scientists investigate the connection between material microstructures and properties to extend fundamental knowledge. On the other hand, textile scientists are grounded in the practical needs of scaling up production. Manufacturing smart textiles at scale continues to be a challenge [2,14,15,24,97,106]. While textiles can be produced at high production speeds [26], smart textile manufacturing depends on the techniques needed to achieve functionality [14] and cost [11,27,51,114]. For example, fiber extrusion is better suited to scale [100,114]. Production speed depends on how automated the method is [27]; notably, integrating textiles and electronics remains mostly manual to this date [13,22,43,65,97]. “Fab labs” [2], robotic processes [7], and desktop robotic 3D printers [93] may support high volume custom manufacturing.

### 3.3. Quality and Testing Standards

It is notable that no smart textile testing or qualification standards exist [13,22], including no standards for output testing [27], wearability, stability, washability, and energy efficiency [82]. In fact, textiles and electronics have separate regulatory requirements [14]. The International Electrotechnical Commission (IEC) TC 124, “Wearable Electronic Devices and Technologies”, is working on standards for materials (electrochromic films, conductive yarns), components (electrical resistance testing, strain sensors testing, snap buttons/modular), and devices (garment washability, step counting, finger movement on glove, skin temperature, burn safety and “Smart Body Area Network”) [115]. Additionally, support for consumer performance testing, e.g., in store changing rooms, is needed [11].

### 3.4. Prototyping

The ease of smart textile prototyping [13] depends on the availability of microcontroller platforms such as Arduino; sensors and interconnects made from conductive textiles and inks; and small ready to use sensors.

The two major toolkits, Arduino Lilypad and Adafruit (FLORA or GENNA), include traditional electronic elements, conductive thread, and a microprocessor [116,117]. Toolkits are used by academic, do-it-yourself, and commercial practitioners and informed by academic research [1]. Toolkits are open-ended with “wide walls” and low barriers to entry, costing less than USD 50 [1,11,116,117]. Smart textile toolkits round and “feminize” traditional electronics to fit textiles and have influenced traditional electronics kits to contain larger holes for connections [1]. Toolkits can be enhanced with other commercially available materials (Table 4).

Yet, toolkits have limitations. Toolkits only support electronic textiles built by attaching hard electronics to soft textiles. The kits do not include fabric or disclose the properties of “conductive thread” [1]. Kits provide compatible connections and components to launch entry level investigators, i.e., hobbyists of the field [1]. Future kits should address the gap between packaged toolkits and cutting-edge research. Moreover, future kits could use interaction and positive aesthetics to encourage material expertise, network solutions, and component design [1]. This would promote education for scholarly research and training of the workforce for manufacturing and entrepreneurship.

### 3.5. Standardized Electronic Textile

A standard textile with built-in interconnects to which components are attached is called a “universal smart textile system” [53], “simulated nervous system of sensors” [54], connected intelligent textile [11], body area network [11], fabric circuit board [114], and “second skin” [103]. A standardized electronic textile would replace custom development; it would support faster development cycles by being mass producible, agnostic to end-use, a customizable framework for interconnects, and for testing [12,13,53,114]. The standardized electronic textile would need to be washable, have redundant flexible connections for power and data transmission, and a dense layout of sensor connection nodes to support tens if not hundreds of reprogrammable sensors [53,103,114]. Piezo, conductive, and optical lines could support non-textile inertial sensors and electrodes [53]. Alternatively, the textiles—fibers, threads, yarns—could behave as electronic components [103].

However, a standardized electronic textile is challenging to make and use. First, defining and making connection points between sensors and wires requires flexible conductive wiring, e.g., by looped stitch interconnects [53,122]. Second, device powering requires continuous power generation, such as by hybrid energy harvesting [79]. Third, cutting and sewing without destroying connections, fashionable designs for universal sizes and styles, and moisture handling [53] must all be resolved before a standardized electronic textile can be sold.

### 3.6. Commercial Products

The commercialization of smart textiles remains difficult [17]. The global wearable market, which includes smart apparel, is expected to grow five-fold between 2016 and 2026 with about half the market going to global market leaders—Apple, Xiaomi, Fitbit, Huawei, and Garmin [11]. While smart textiles lag behind gadgets, i.e., Fitbit and Apple Smart Watch [11,103], smart eyewear has already switched formats to industrial (Google Glass 2.0, [123]) or contact lens (Mojo vision, [124]). Top tech and fashion brands have teamed up to make smart textile apparel, cashing in on brand recognition; notable players include Google (with Levi), Apple, Samsung, Intel, Ralph Lauren, Polar, and Under Armor [11]. Although the smart textile market is expected to grow [78], products continue to struggle.

Why is this? Startups may have rushed to be “first to market” and capitalize on “tech-fetishism” [106]. Smart textile startups can quickly prototype products, which causes high competition and market noise. Often, products fail to meet expectations or live up to the hype. Most new technology products fail to convert the early adopters and tech evangelists into mass market appeal [125]. Academic research has low technology readiness levels (TRLs 1–2), while commercial products have high TRLs (6–9) [11]. Government labs, with mid TRLs (3–5) [113], are instrumental in moving tech from academia to commercialization by standards’ development. Finally, hidden risks, such as liability and lawsuits for medical claims, may block continued success [126].

Commercial smart textiles can be divided into sensor fabrics and heating garments. The oldest commercial sensor fabric on the market, the Reima Cyberia survival suit, launched in 2000, has GPS, a hydrometer, thermometer, and embroidered electrodes [11]. Motorbike suits (Dainese D-AIR, [22,127]), safety shoes (Izome, [103]), running insoles (Arion, [128]), and health garments (Myant [129], Texis Sense for Life [130], Numetrex, SmartLife HealthVest, and Exmovere Exmobaby [3,113]) are available. Heating garments tend to be for sport/athletic applications. Commercial resistive heating products include Blaze Wear [131] and Team USA Olympic heated jackets [132]. The Mide SmartSkinTM diving swimsuit [6] and Nike “Sphere React Shirt” [6] used responsive hydrogels or vents to regulate temperature, although neither is available for sale. Tibtech produces conductive heating yarns and fabrics for industrial de-icing [103,133]. In summary, while resistive style heating is available, non-electronic adaptive thermal comfort products have yet to take hold.

## 4. Outlook

### 4.1. New Textile Production Methods

New textile production methods include thicker digital printing (dispenser printing), a 3D printing fabric, motorized stitch gathering, and laser cut folding. Dispenser printing (DP) performs computer-aided printing to deposit an ink thickness similar to screen printing after curing, i.e., a much thicker layer of metal than digital inkjet printing [134]. Three-dimensional printers produce smart textile objects by layering cut off-the-shelf felt or conductive fabric bonded with heat fusible adhesive (Heat-n-Bond^TM^) [93]. The placement of cuts controls the deformation properties, and conductive fabric can be used to make touch sensors, circuit paths, or interlayer vias [93]. A new–old method sews seams onto fabric, which, when pulled, change the textile shape (lateral gather (pleat), horizontal gather (bend), or diagonal simple/curved gather) to make an adjustable skirt length or self-opening curtain when attached to sensors and a motor [72]. A conformable shoe sole with foam-like compliance was made by Tachi-Miura polyhedral origami folding of PP film on a 3D printed plastic guide reinforced with cotton thread [135]. These reframed techniques provide greater responsivity.

### 4.2. A Smart Textiles Journal

Currently, no “e-Textile” or “Smart Textile” journal exists. Researchers publish in discipline specific journals; in fact, most publications are outside of textile journals (88%) even though almost a third (29%) of the researchers have a textiles background or affiliation. Other interdisciplinary fields have a shared journal, e.g., “Additive Manufacturing”, launched in 2014. An interdisciplinary field requires interdisciplinary information sharing, e.g., user experience or tech adoption best practices into materials or electrical engineering papers. A smart textiles journal should exist; some of the proposed journal areas with possible fields that can contribute to e-textiles are displayed in Table 5.

## 5. Conclusions

In conclusion, the smart textiles field is both mature and up-and-coming. E-textiles contain multiple scales—fibers, threads or yarns, fabrics, garments, ensembles, and assemblies of textile wearers—across which smart interactions could be designed [5,7]. Surveys of each component have highlighted the various mechanisms utilized to “sense” and “actuate” while requiring some form of “power” that are “interconnected”. Applications are surveyed, as well as the current limitations facing the e-textiles field, such as their commercialization, standardization, prototyping, and highly interdisciplinary nature. Implementing interactions designed for specific applications and wearers will help academic research gain enough traction to leave the lab. More well-informed and coordinated interdisciplinary collaborations are also crucial to solve the remaining challenges such as developing a standardized electronic textile, battery-less stimuli responsive garments, and sustainable manufacturing methods. The material palette is limited solely by the researcher’s creativity and encompasses polymers–metals–ceramics and fibers–films–fabrics. Perhaps the most exciting, underdeveloped application area is textiles that make virtual reality a tactile reality.

## Figures and Tables

**Table 1:** Conductive materials used in smart textiles, their electrical conductivity and percolation threshold.

Material	Conductivity	Percolation Threshold *
Copper	5.87 × 10^7^ S/m [30]	37% volume [31]
Gold	4.42 × 10^7^ S/m [30]	39% volume for co-sputtered gold/poly(tetrafluoroethylene) (PTFE) film [32]
Silver	6.21 × 10^7^ S/m [30]	7–16 vol% in polyvinylidene difluoride (PVDF) [33]
Carbon Black	10^1^–10^4^ S/m [34]	0.58 wt% in polyethylene terephthalate (PET) [35]
Graphene	6.0 × 10^5^ S/m (isolated) [36]	0.47 vol% in PET [37]
Carbon Nanotube (CNT)	10^6^–10^7^ S/m [38]	1.2 wt% (CNT in PVDF) [39]
Ionic Liquid	1.3 × 10^−2^ –1.4 × 10^0^ S/m [40]	Decreased percolation threshold of graphene in urethane from 3.21 wt% to 1.99 wt% due to better graphene dispersion [41]
PVDF	10^−2^ S/m [42]	N/A—typically used as a matrix

* Percolation thresholds given are best available or purely illustrative. Percolation depends on the polymer matrix, particle size and dimensions, and the dispersion quality.

**Table 2:** Sensors used in electronic textile.

Type	Material	Format	Mechanism	Ref.
Motion	Rigid electronic	Inertial motion capture	magnetometers, accelerometers, and gyroscopes	[56]
	Bending sensor	Optical fiber (Bragg grating)	Optics	[22]
	Carbon black dip-coated co-polyester elastomer or spandex filament	Sensors attached to t-shirt	Strain-induced disruption and connection of conductive pathways affects electrical resistance (piezoresistive).	[56]
	Machine knit elastomeric and conductive (80% polyester, 20% stainless steel) multifilament yarns	Rehabilitation glove	Strain affects contact resistance (Holm’s contact theory)	[44]
	Flexible, non-crocking reduced graphene oxide fabric through dip coating and nickel electroless plating	Strain sensor	Strain affects resistance	[54]
	Conductive polymer filaments	Strain sensor	resistance change in paired (stretched/relaxed) sensors	[59]
	Hand-knit together cotton yarn and wire	Inductor coils	Increasing radius increases inductance	[60]
Physiology	Electrode	Carbon or conductive yarns (stainless steel)	Change in resistance due to stimuli	[50]
	highly conductive, nitrogen-doped working electrodes	carbonized or graphitized woven silk fabric	Circuit converts signal into data for mobile display Current: glucose, lactate Potential: sodium, potassium	[61]
	“wet” electrode (sweat is electrolyte)	conductive knit fabric (Shieldex Fabric by Statex) knife-coated with a conductive paste	Measure Biopotential	[62]
	(EEG) sensor	layers of conductive and sweat absorbent fabrics	Measure Biopotential (~100 μV)	[63]
	Blood oxygenation	Rigid electronics	oxygenated and deoxygenated hemoglobin absorb different amounts of light	[52]
	Antennas	Conductive fabric attached to silicone rubber substrate	Resonance frequency interference between antennas corresponds to brain atrophy and lateral ventricle enlargement	[64]
Environment	Temperature sensors	printing conductive inks	change resistance in response to temperature [22]	[65]
	Temperature sensors	weaving electronic strips into textile	change resistance in response to temperature [22]	[65]
	Temperature sensors	encapsulating temperature sensor in yarn core	change resistance in response to temperature [22]	[65]
	Humidity sensor	poly(3,4-ethylenedioxythiophene) polystyrene sulfonate (PEDOT:PSS) on a substrate of polyacrylonitrile nanofibers	materials change conductivity in response to moisture	[22]
	flexible ammonia sensor	cotton yarn coated with carbon nanotube ink	exposure to chemical changes resistance, “chemiresistor”	[66]
	multimodal	“Carbon Nanotube Paint” coated degummed silk fiber	electrical resistance changes with stimuli	[67]

**Table 3:** Actuators used in electronic textile.

Type	Material	Mechanism	Ref.
Speakers	sandwiching layers of piezoelectric polyvinylidene difluoride (PVDF) film/zinc oxide pillars on fabrics printed with conductive inks	Electronics	[14]
Mechanical actuator	Motorized seams sewn onto fabric	pulling seam changes the textile shape	[72]
Sensor/actuator	sewing, couching, shape memory alloy fiber onto fabric and painting conductive ink	strain sensor which responds to cutting, heating, or pressure	[73]
Mechanical actuator	conductive textiles cut, coated, and laminated	Electro-adhesive actuators and dielectric elastomer actuators	[74]
Display	knit or woven electroluminescent fibers	electrically controlled fabric visual display	[75]
Display	tactile enhanced fabric display	electrostatically actuated with electrodes	[76]
Vibrotactile displays	film	tactile elements operate independently based on mechanical resonance frequency	[77]

**Table 4:** Commercial materials for prototyping e-textiles.

Component	Company	Description	Ref.
Sensor + Actuator + Interconnects	Dupont	Stretchable inks for wearables: carbon, silver, or silver/silver chloride conductor encapsulant material	[118]
Sensor + Actuator + Interconnects	FabInks	Smart fabric inks (ultraviolet (UV) or thermal cured) interface, encapsulation, conductor, dielectric, piezoelectric, thermochromic, electrode, sacrificial	[119]
Sensor	Primo1D e-Thread	RFID yarn: yarn twisted around chip to hide it [103]	[120]
Sensor + Actuator + Interconnects	Bekaert Fibre Technologies	Conductive yarn 1–80 μm diameter, 8–14 μm fibers	[26,121]
Actuator Fabric	Thermolactyl	Triboelectric heating fiber	[103]

**Table 5:** Smart textile research publication by disciplines.

Journal Focus	Purpose	Disciplines
Prototypes of Wearables	Focused on e-textile system (power, sensing/actuating, connections).	Electrical and computer engineering, information systems
User experience/ adoption of tech	Voice of the customer, market analysis	Business, marketing, design, computer–human interface (CHI), psychology, philosophy
Materials processing	Material properties and interactions, integration into a textile or a wearable medium	Materials science, chemical engineering, mechanical engineering, plastics engineering, textile sciences

## Data Availability

Not applicable.

## References

[1] Posch I., Stark L., Fitzpatrick G. (2019). eTextiles: Reviewing a Practice through its Tool/Kits. Proceedings of the 2019 International Symposium on Wearable Computers (ISWC ‘19).

[2] Cutcher-Gershenfeld J., Gershenfeld A., Gershenfeld N. (2008). Digital Fabrication and the Future of Work. Perspect. Work.

[3] Hassib M., Khamis M., Schneegass S., Shirazi A.S., Alt F. Investigating User Needs for Bio-sensing and Affective Wearables. Proceedings of the CHI 2016 Late-Breaking Work: Designing Interactive Systems.

[4] Mossé A. (2018). Gossamer timescapes: A design-led investigation into electro-active and light responsive textiles for the home. Smart Mater. Struct..

[5] Herbert K.M., Schrettl S., Rowan S.J., Weder C. (2017). 50th Anniversary Perspective: Solid-State Multistimuli, Multiresponsive Polymeric Materials. Macromolecules.

[6] Hu J., Meng H., Li G., Ibekwe S.I. (2012). A review of stimuli-responsive polymers for smart textile applications. Smart Mater. Struct..

[7] Heinzel T., Hinestroza J.P. (2020). Revolutionary Textiles: A Philosophical Inquiry on Electronic and Reactive Textiles. Des. Issues.

[8] Karana E., Nimkulrat N., Giaccardi E., Niedderer K., Fan J.-N. (2019). Alive. Active. Adaptive: Experiential Knowledge and Emerging Materials. Int. J. Des..

[9] Ghahremani M., Latifi M., Babaei M. (2017). Simulation of conductivity made by inkjet-printed silver tracks in E-textiles with different weave Patterns. J. Ind. Text..

[10] Ferri A., Plutino M.R., Rosace G. (2019). Recent trends in smart textiles: Wearable sensors and drug release systems. AIP Conf. Proc..

[11] Fernández-Caramés T.M., Fraga-Lamas P. (2018). Towards The Internet of Smart Clothing: A Review on IoT Wearables and Garments for Creating Intelligent Connected E-Textiles. Electronics.

[12] Ray S., Park J., Bhunia S. (2016). Wearables, Implants, and Internet of Things: The Technology Needs in the Evolving Landscape. IEEE Trans. Multi-Scale Comput. Syst..

[13] Jansen K.M.B. How to Shape the Future of Smart Clothing. Proceedings of the UbiComp/ISWC 2019—Adjunct Proceedings of the 2019 ACM International Joint Conference on Pervasive and Ubiquitous Computing and Proceedings of the 2019 ACM International Symposium on Wearable Computers.

[14] Yang K., Isaia B., Brown L.J., Beeby S. (2019). E-Textiles for Healthy Ageing. Sensors.

[15] Stewart R. (2019). Cords and Chords: Exploring the Role of E-Textiles in Computational Audio. Front. ICT.

[16] Cherenack K., van Pieterson L. (2012). Smart textiles: Challenges and opportunities. J. Appl. Phys..

[17] Sanchez V., Walsh C.J., Wood R.J. (2021). Textile Technology for Soft Robotic and Autonomous Garments. Adv. Funct. Mater..

[18] Tabor J., Chatterjee K., Ghosh T.K. (2020). Smart Textile-Based Personal Thermal Comfort Systems: Current Status and Potential Solutions. Adv. Mater. Technol..

[19] Loke G., Yan W., Khudiyev T., Noel G., Fink Y. (2020). Recent Progress and Perspectives of Thermally Drawn Multimaterial Fiber Electronics. Adv. Mater..

[20] Ruckdashel R.R., Venkataraman D., Park J.H. (2021). Smart textiles: A toolkit to fashion the future. J. Appl. Phys..

[21] Nanjappan V., Shi R., Liang H.-N., Lau K.K.-T., Yue Y., Atkinson K. (2019). Towards a Taxonomy for In-Vehicle Interactions Using Wearable Smart Textiles: Insights from a User-Elicitation Study. Multimodal Technol. Interact..

[22] Gonçalves C., da Silva A.F., Gomes J., Simoes R. (2018). Wearable E-Textile Technologies: A Review on Sensors, Actuators and Control Elements. Inventions.

[23] Komolafe A., Torah R., Tudor M., Beeby S. (2019). Modelling Reliable Electrical Conductors for E-Textile Circuits on Polyimide Filaments. Multidiscip. Digit. Publ. Inst. Proc..

[24] Tasnim F., Sadraei A., Datta B., Khan M., Choi K.Y., Sahasrabudhe A., Gálvez T.A.V., Wicaksono I., Rosello O., Nunez-Lopez C. (2018). Towards personalized medicine: The evolution of imperceptible health-care technologies. Foresight.

[25] Corchia L., Monti G., de Benedetto E., Tarricone L. A Chipless Humidity Sensor for Wearable Applications. Proceedings of the IEEE International Conference on RFID Technology and Applications (RFID-TA).

[26] Borazan I., Kaplan M., Uzumcu M.B. Utilization of Metallic Fibers in Textiles. Proceedings of the 2nd International Congress of Innovative Textiles.

[27] Dong K., Peng X., Wang Z.L. (2019). Fiber/Fabric-Based Piezoelectric and Triboelectric Nanogenerators for Flexible/Stretchable and Wearable Electronics and Artificial Intelligence. Adv. Mater..

[28] Gao Y., Cho J.H., Ryu J., Choi S. (2020). A scalable yarn-based biobattery for biochemical energy harvesting in smart textiles. Nano Energy.

[29] Alharbi S., Chaudhari S., Inshaar A., Shah H., Zou C., Harne R.L., Kiourti A. (2018). E-Textile Origami Dipole Antennas With Graded Embroidery for Adaptive RF Performance. IEEE Antennas Wirel. Propag. Lett..

[30] Conductive Materials, Metals and Stainless Steels Properties Table: Tibtech. https://www.tibtech.com/conductivite.php?lang=en_US.

[31] Wang J., Chang A.S., Sherfield S.N., Golobic A.M., Hunter S.L., Duoss E.B., Matthews M.J. Electrical Properties of Copper Loaded Polymer Composites. Proceedings of the SPIE Smart Structures + Nondestructive Evaluation.

[32] Liu F., Shang S., Duan Y., Li L. (2012). Electrical and optical properties of polymer-Au nanocomposite films synthesized by magnetron cosputtering. J. Appl. Polym. Sci..

[33] Deepa K., Gopika M., James J. (2013). Influence of matrix conductivity and Coulomb blockade effect on the percolation threshold of insulator–conductor composites. Compos. Sci. Technol..

[34] Pantea D., Darmstadt H., Kaliaguine S., Sümmchen L., Roy C. (2001). Electrical conductivity of thermal carbon blacks: Influence of surface chemistry. Carbon.

[35] Choi H.-J., Kim M.S., Ahn D., Yeo S.Y., Lee S. (2019). Electrical percolation threshold of carbon black in a polymer matrix and its application to antistatic fibre. Sci. Rep..

[36] Du X., Skachko I., Barker A., Andrei E.Y. (2008). Approaching ballistic transport in suspended graphene. Nat. Nanotechnol..

[37] Zhang H.-B., Zheng W.-G., Yan Q., Yang Y., Wang J.-W., Lu Z.-H., Ji G.-Y., Yu Z.-Z. (2010). Electrically conductive polyethylene terephthalate/graphene nanocomposites prepared by melt compounding. Polymer.

[38] Choi J., Zhang Y. Single-Double Multi-Walled Carbon Nanotubes.

[39] Wang S.H., Wan Y., Sun B., Liu L.Z., Xu W. (2014). Mechanical and electrical properties of electrospun PVDF/MWCNT ultrafine fibers using rotating collector. Nanoscale Res. Lett..

[40] Galiński M., Lewandowski A., Stępniak I. (2006). Ionic liquids as electrolytes. Electrochim. Acta.

[41] Aranburu N., Otaegi I., Guerrica-Echevarria G. (2019). Using an Ionic Liquid to Reduce the Electrical Percolation Threshold in Biobased Thermoplastic Polyurethane/Graphene Nanocomposites. Polymers.

[42] Puértolas J., García-García J., Pascual F., González-Domínguez J., Martínez M., Ansón-Casaos A. (2017). Dielectric behavior and electrical conductivity of PVDF filled with functionalized single-walled carbon nanotubes. Compos. Sci. Technol..

[43] Micus S., Kirsten I., Haupt M., Gresser G.T. (2020). Analysis of Hot Bar Soldering, Insulation Displacement Connections (IDC), and Anisotropic Conductive Adhesives (ACA), for the Automated Production of Smart Textiles. Sensors.

[44] Ayodele E., Zaidi S., Zhang Z., Scott J., Kong Q., McLernon D. Weft Knit Smart Data Glove. Proceedings of the IEEE 16th International Conference on Wearable and Implantable Body Sensor Networks (BSN).

[45] Stark L. Data Vows: Reimagining Ritual through eTextile Practice. Proceedings of the ISWC ‘17: Proceedings of the 2017 ACM International Symposium on Wearable Computers.

[46] Baum T.C., Ziolkowski R.W., Ghorbani K., Nicholson K.J. (2016). Embroidered Active Microwave Composite Preimpregnated Electronics—Pregtronics. IEEE Trans. Microw. Theory Tech..

[47] Orth M. (2002). Defining Flexibility and sewability in conductive yarns. Proc. Mater. Res. Soc. Symp..

[48] Castano L.M., Flatau A.B. (2014). Smart fabric sensors and e-textile technologies: A review. Smart Mater. Struct..

[49] Sargolzaeiaval Y., Ramesh V.P., Neumann T.V., Misra V., Vashaee D., Dickey M.D., Öztürk M.C. (2020). Flexible thermoelectric generators for body heat harvesting—Enhanced device performance using high thermal conductivity elastomer encapsulation on liquid metal interconnects. Appl. Energy.

[50] Coyle S., Lau K.-T., Moyna N., O’Gorman D., Diamond D., di Francesco F., Costanzo D., Salvo P., Trivella M.G., de Rossi D.E. (2010). BIOTEX—Biosensing Textiles for Personalised Healthcare Management. IEEE Trans. Inf. Technol. Biomed..

[51] Lemey S., Agneessens S., Rogier H. (2018). Wearable Smart Objects. IEEE Microw. Mag..

[52] Agrawal R., Koteswarapavan C., Kaushik N., Matre P., Shukla P. (2018). Smart actuators for innovative biomedical applications. Applied Microbiology and Bioengineering: An Interdisciplinary Approach.

[53] Nesenbergs K., Selavo L. Smart textiles for wearable sensor networks: Review and early lessons. Proceedings of the 2015 IEEE International Symposium on Medical Measurements and Applications (MeMeA) Proceedings.

[54] Chen Y., Xu B., Gong J., Wen J., Hua T., Kan C.-W., Deng J. (2019). Design of High-Performance Wearable Energy and Sensor Electronics from Fiber Materials. Appl. Mater. Interfaces.

[55] Bahadır S.K., Atalay Ö., Kalaoglu F., Mitilineos S.A., Vassiliadis S. (2018). High Frequency Attenuation Characterization of Knitted E-Textile Structures. IOP Conf. Ser. Mater. Sci. Eng..

[56] Rezaei A., Cuthbert T.J., Gholami M., Menon C. (2019). Application-Based Production and Testing of a Core–Sheath Fiber Strain Sensor for Wearable Electronics: Feasibility Study of Using the Sensors in Measuring Tri-Axial Trunk Motion Angles. Sensors.

[57] Shui X., Chung D.D.L. (1996). A piezoresistive carbon filament polymer-matrix composite strain sensor. Smart Mater. Struct..

[58] McLaren R., Joseph F., Baguley C., Taylor D. (2016). A review of e-textiles in neurological rehabilitation: How close are we?. J. NeuroEngineering Rehabil..

[59] Strohmeier P.R., Vertegaal R., Girouard A. With a flick of the wrist: Stretch sensors as lightweight input for mobile devices. Proceedings of the 6th International Conference on Tangible and Embedded Interaction 2012.

[60] Fobelets K., Thielemans K., Mathivanan A., Papavassiliou C. (2019). Characterization of Knitted Coils for e-Textiles. IEEE Sens. J..

[61] He W., Wang C., Wang H., Jian M., Lu W., Liang X., Zhang X., Yang F., Zhang Y. (2019). Integrated textile sensor patch for real-time and multiplex sweat analysis. Sci. Adv..

[62] Soroudi A., Hernández N., Wipenmyr J., Nierstrasz V. (2019). Surface modification of textile electrodes to improve electrocardiography signals in wearable smart garment. J. Mater. Sci. Mater. Electron..

[63] Shu L., Xu T., Xu X. (2019). Multilayer Sweat-Absorbable Textile Electrode for EEG Measurement in Forehead Site. IEEE Sens. J..

[64] Saied I.M., Chandran S., Arslan T. (2019). Integrated Flexible Hybrid Silicone-Textile Dual-Resonant Sensors and Switching Circuit for Wearable Neurodegeneration Monitoring Systems. IEEE Trans. Biomed. Circuits Syst..

[65] Komolafe A., Torah R., Nunes-Matos H., Tudor M., Beeby S. Integration of temperature sensors in fabrics. Proceedings of the 2019 IEEE International Conference on Flexible and Printable Sensors and Systems (FLEPS).

[66] Han J.-W., Kim B., Li J., Meyyappan M. (2013). A carbon nanotube based ammonia sensor on cotton textile. Appl. Phys. Lett..

[67] Ye C., Ren J., Wang Y., Zhang W., Qian C., Han J., Zhang C., Jin K., Buehler M.J., Kaplan D.L. (2019). Design and Fabrication of Silk Templated Electronic Yarns and Applications in Multifunctional Textiles. Matter.

[68] González E., Shepherd L.M., Saunders L., Frey M.W. (2016). Surface Functional Poly(lactic Acid) Electrospun Nanofibers for Biosensor Applications. Materials.

[69] Haque M. (2019). Nano Fabrics in the 21st century: A review. Asian J. Nanosci. Mater..

[70] Soroudi A., Hernandez N., Berglin L., Nierstrasz V. (2019). Electrode placement in electrocardiography smart garments: A review. J. Electrocardiol..

[71] Drew B. (2011). Standardization of electrode placement for continuous patient monitoring: Introduction of an assessment tool to compare proposed electrocardiogram lead configurations. J. Electrocardiol..

[72] Kono T., Watanabe K. Filum: A Sewing Technique to Alter Textile Shapes. Proceedings of the UIST’17 Adjunct Publication of the 30th Annual ACM Symposium.

[73] Buckner T.L., Bilodeau R.A., Ki S.Y., Kramer-Bottiglio R. (2020). Roboticizing fabric by integrating functional fibers. Proc. Natl. Acad. Sci. USA.

[74] Guo J., Xiang C., Helps T., Taghavi M., Rossiter J. Electroactive textile actuators for wearable and soft robots. Proceedings of the 2018 IEEE International Conference on Soft Robotics (RoboSoft).

[75] Zhang Z., Cui L., Shi X., Tian X., Wang D., Gu C., Chen E., Cheng X., Xu Y., Hu Y. (2018). Textile Display for Electronic and Brain-Interfaced Communications. Adv. Mater..

[76] Sahoo D.R., Hornbæk K., Subramanian S. Deepak Ranjan Sahoo1, TableHop: An Actuated Fabric Display Using Transparent Electrodes. Proceedings of the 2016 CHI Conference on Human Factors in Computing Systems (CHI ‘16).

[77] Mohammadia A., Abdelkhalekb M., Sadrafsharia S. (2020). Resonance frequency selective electromagnetic actuation for high-resolution vibrotactile displays. Sens. Actuators A Phys..

[78] Kiourti A. Textile-Based Flexible Electronics for Wearable Applications: From Antennas to Batteries. Proceedings of the 2018 2nd URSI Atlantic Radio Science Meeting (AT-RASC).

[79] García Núñez C., Manjakkal. L., Dahiya R. (2019). Energy autonomous electronic skin. NPJ Flex. Electron..

[80] Lemey S., Agneessens S., Rogier H. Textile SIW Antennas as Hybrid Energy Harvesting and Power Management Platforms. Proceedings of the 45th European Microwave Conference.

[81] Komolafe A., Wagih M., Valavan A., Ahmed Z., Stuikys A., Zaghari B. (2019). A Smart Cycling Platform for Textile-Based Sensing and Wireless Power Transfer in Smart Cities. Multidiscip. Digit. Publ. Inst. Proc..

[82] Chen G., Li Y., Bick M., Chen J. (2020). Smart Textiles for Electricity Generation. Chem. Rev..

[83] Nozariasbmarz A., Collins H., Dsouza K., Polash M.H., Hosseini M., Hyland M., Liu J., Malhotra A., Ortiza F.M., Mohaddes F. (2020). Review of wearable thermoelectric energy harvesting: From body temperature to electronic systems. Appl. Energy.

[84] Toivola M., Ferenets M., Lund P., Harlin A. (2009). Photovoltaic fiber. Thin Solid Film..

[85] Park N.-G. (2015). Perovskite solar cells: An emerging photovoltaic technology. Materialstoday.

[86] Rein M., Favrod V.D., Hou C., Khudiyev T., Stolyarov A., Cox J., Chung C.-C., Chhav C., Ellis M., Joannopoulos J. (2018). Diode fibres for fabric-based optical communications. Nature.

[87] Yan W., Page A., Nguyen-Dang T., Qu Y., Sordo F., Wei L., Sorin F. (2019). Advanced Multimaterial Electronic and Optoelectronic Fibers and Textiles. Adv. Mater..

[88] Lee M.R., Eckert R., Forberich K., Dennler G., Brabec C.J., Gaudiana R.A. (2009). Solar Power Wires Based on Organic Photovoltaic Materials. Science.

[89] Lund A., Rundqvist K., Nilsson E., Yu L., Hagström B., Müller C. (2018). Energy harvesting textiles for a rainy day: Woven piezoelectrics based on melt-spun PVDF microfibres with a conducting core. NPJ Flex. Electron..

[90] Posch I., Kurbak E. Crafted Logic: Towards Hand-Crafting a Computer. Proceedings of the CHI’16 Extended Abstracts.

[91] Shi Q., Zhang Z., Chen T., Lee C. (2019). Minimalist and multi-functional human machine interface (HMI) using a flexible wearable triboelectric patch. Nano Energy.

[92] Xu P.A., Mishra A.K., Bai H., Aubin C.A., Zullo L., Shepherd R.F. (2019). Optical lace for synthetic afferent neural networks. Sci. Robot..

[93] Peng H., Mankoff J., Hudson S.E., McCann J. A Layered Fabric 3D Printer for Soft Interactive Objects. Proceedings of the CHI 2015, Crossings, Design and 3D Object Fabrication.

[94] Loughborough University London (2019). Textile Intersections. In Intersections Exhibition. https://cfpr.uwe.ac.uk/textile-intersections-exhibition/.

[95] Dong W., Xiao L., Hu W., Zhu C., Huang Y., Yin Z. (2017). Wearable human–machine interface based on PVDF piezoelectric sensor. Trans. Inst. Meas. Control.

[96] Seth R. (2016). Wearable Wireless Hmi Device. United States of. America Patent.

[97] Poupyrev I., Gong N.-W., Fukuhara S., Karagozler M.E., Schwesig C., Robinson K.E. (2016). Project Jacquard: Interactive Digital Textiles at Scale.

[98] Khoshkava V., Cruz-Hernandez J.M. (2018). Bifunctional Fiber for Combined Sensing and Haptic Feedback. USA Patent.

[99] Gorgutsa S., Gu J.F., Skorobogatiy M. (2012). A woven 2D touchpad sensor and a 1D slide sensor using soft capacitor fibers. Smart Mater. Struct..

[100] Kapoor A., McKnight M., Chatterjee K., Agcayazi T., Kausche H., Bozkurt A., Ghosh T.K. (2019). Toward Fully Manufacturable, Fiber Assembly–Based Concurrent Multimodal and Multifunctional Sensors for e-Textiles. Adv. Mater. Technol..

[101] Mikkonen J., Townsend R. (2019). Frequency-Based Design of Smart Textiles. Proceedings of the CHI Conference on Human Factors in Computing Systems Proceedings (CHI 2019).

[102] Zolyomi A., Gotfrid T., Shinohara K. (2019). Socializing via a Scarf: Individuals with Intellectual and Developmental Disabilities Explore Smart Textiles.

[103] Paret D., Crégo P. (2019). Wearables, Smart Textiles & Smart Apparel.

[104] Rantakari J., Inget V., Colley A., Häkkila J. Charting Design Preferences on Wellness Wearables. Proceedings of the 7th Augmented Human International Conference 2016 (AH ’16). Association for Computing Machinery.

[105] Moradi B., Fernandez-Garcia R., Gil I. (2019). Effect of smart textile metamaterials on electromagnetic performance for wireless body area network systems. Text. Res. J..

[106] Bryan-Kinns N., Wu Y., Liu S., Baker C. WEAR Sustain Network: Ethical and Sustainable Technology Innovation in Wearables and Etextiles. Proceedings of the IEEE Games, Entertainment, Media Conference (GEM).

[107] Lam C.-S., Ramanathan S., Carbery M., Gray K., Vanka K.S., Maurin C., Bush R., Palanisami T. (2018). A Comprehensive Analysis of Plastics and Microplastic Legislation Worldwide. Water Air Soil Pollut..

[108] Mahmoud K.H.M., Salam S.H.A., El-Hadi H. (2020). Designing Smart Textiles Prints with Interactive Capability. J. Des. Sci. Appl. Arts.

[109] Lee J.J., Hammer J. (2011). Gamification in Education: What, How, Why Bother?. Acad. Exch. Q..

[110] Pal D., Vanijja V., Arpnikanondt C., Zhang X., Papasratorn B. (2019). A Quantitative Approach for Evaluating the Quality of Experience of Smart-Wearables From the Quality of Data and Quality of Information: An End User Perspective. IEEE Access.

[111] Latour B., Woolgar S. (1986). Laboratory Life: The Construction of Scientific Facts.

[112] Alvesson M., Sandberg J. (2011). Generating Research Questions Through Problematization. Acad. Manag. Rev..

[113] Simon C., Potter E., McCabe M., Baggerman C. (2010). Smart Fabrics Technology Development.

[114] Jansen K. Smart textiles: How electronics merge into our clothing. Proceedings of the 20th International Conference on Thermal, Mechanical and Multi-Physics Simulation and Experiments in Microelectronics and Microsystems (EuroSimE).

[115] International Electrotechnical Commission. https://www.iec.ch/dyn/www/f?p=103:30:5818194881611::::FSP_ORG_ID,FSP_LANG_ID:20537,25.

[116] Sparkfun. Lilypad Protosnap Plus Kit. Sparkfun. https://www.sparkfun.com/products/12922.

[117] Adafruit. Wearables. Adafruit. https://www.adafruit.com/category/65.

[118] du Pont. Stretchable Inks for Wearable Electronics. du Pont. https://www.dupont.com/products/stetchable-inks-for-wearable-electronics.html.

[119] Smart Fabric Inks. Product. Smart Fabric Inks. http://www.fabinks.com/product/.

[120] Primo1D. The Technology. Primo1D. https://www.primo1d.com/e-thread/the-technology.

[121] Bekaert. Conductive Fibers and Yarns for Smart Textiles. Bekaert. https://www.bekaert.com/en/products/basic-materials/textile/conductive-fibers-and-yarns-for-smart-textiles.

[122] Vieroth R., Löher T., Seckel M., Dils C., Kallmayer C., Ostmann A., Reichl H. Stretchable circuit board technology and application. Proceedings of the 2009 International Symposium on Wearable Computers (ISWC ‘09).

[123] Levy S. Google Glass 2.0 Is a Startling Second Act. Wired. 18 July 2017. https://www.wired.com/story/google-glass-2-is-here/.

[124] Kirsh D. Mojo Vision Developing “Smart” Contact Lens. Mass Device. 17 January 2020. https://www.massdevice.com/mojo-vision-developing-smart-contact-lens/.

[125] Moore G.A. (2006). Crossing the Chasm: Marketing and Selling High-Tech Products to Mainstream Customers.

[126] Newmarker C. 4 Medical Device Industry Trends Affecting Innovation. Medical Design & Outsourcing. 6 January 2020. https://www.medicaldesignandoutsourcing.com/4-medical-device-industry-trends-affecting-innovation/.

[127] Dianese. Space Suits. Dianese. https://www.dainese.com/us/en/technology-innovation/space-suits.html.

[128] Arion. Wearable. Arion. https://www.arion.run/wearable/.

[129] Crotti N. Heraeus Medical Components and Myant Partner on Electrical Sensing for Textiles. Medical Design & Outsourcing. 22 January 2020. https://www.medicaldesignandoutsourcing.com/heraeus-medical-components-and-myant-partner-on-electrical-sensing-for-textiles/.

[130] Texisense. Texisense Corporate Website. Texisense. https://www.texisense.com/.

[131] Blaze Wear. About US: Blaze Wear. Blaze Wear. https://www.blazewear.com/about-us.

[132] Noe R. How Team USA’s Self-Heating Olympic Jackets Work, and a List of the Design Firms That Helped to Create Them. Core77. 12 February 2018. https://www.core77.com/posts/73270/How-Team-USAs-Self-Heating-Olympic-Jackets-Work-and-a-List-of-the-Design-Firms-That-Helped-to-Create-Them.

[133] TIBTECH Innovations. Home: TIBTECH Innovations. TIBTECH Innovations. https://www.tibtech.com/.

[134] Torah R., Wei Y., Grabham N., Li Y., de Vos M., Todorov T., Popov B., Marinov V., Stoyanov S., Todorov V. (2019). Enabling platform technology for smart fabric design and printing. J. Eng. Fibers Fabr..

[135] Calisch S., Gershenfeld N.A. (2018). Kirigami Fabrication of Shaped, Flat-Foldable Cellular Materials Based on the Tachi-Miura Polyhedron.

